# Relationship between nerve conduction studies and the Functional Dexterity Test in workers with carpal tunnel syndrome

**DOI:** 10.1186/s12891-020-03651-1

**Published:** 2020-10-14

**Authors:** Francesco Sartorio, Francesca Dal Negro, Elisabetta Bravini, Giorgio Ferriero, Stefano Corna, Marco Invernizzi, Stefano Vercelli

**Affiliations:** 1 Institute of Veruno, Istituti Clinici Scientifici Maugeri IRCCS, Gattico-Veruno (NO), Italy; 2 Italian Society of Physiotherapy, Florence, Italy; 3 Istituti Clinici Scientifici Maugeri IRCCS, Institute of Tradate (VA), Via Maugeri 4, I-27100 Pavia, Italy; 4 Physical and Rehabilitation Medicine, Department of Health Sciences, University of Eastern Piedmont, Novara, Italy

**Keywords:** Clinical tests, Diagnosis, Dexterity, Rehabilitation, Occupational therapy

## Abstract

**Background:**

Dexterity impairments caused by carpal tunnel syndrome (CTS) make working and daily activities challenging. We aimed to investigate: i) the relationship between dexterity and nerve conduction studies (NCS) in workers with classic symptoms presentation; ii) the ability of the Functional Dexterity Test (FDT) to discriminate different levels of CTS severity as classified by NCS; iii) the diagnostic accuracy of a clinical battery composed of the FDT, Phalen’s test and Tinel’s sign.

**Methods:**

In a convenience sample of individuals diagnosed with CTS, we correlated FDT net scores with the NCS-based classification by means of Spearman’s (rho) test. Discriminative ability of the FDT was assessed by ANOVA, and a ROC curve determined cutoff thresholds. Sensitivity, specificity, and likelihood ratios (LRs) were used to investigate the diagnostic accuracy of the clinical battery.

**Results:**

Data from 180 hands were collected. The FDT was significantly correlated (rho = 0.25, *p* <  0.001) with NCS. The FDT was able to discriminate subjects with severe/extreme NCS findings, and two thresholds (0.29–0.36) were identified. Adding the FDT to the provocative tests improved the overall diagnostic accuracy (specificity: 0.97, CI_95%_ 0.83–0.99; LR+: 14.49, CI_95%_ 2.09–100.53).

**Conclusions:**

Sensorimotor impairments related to CTS can affect hand dexterity. The FDT discriminated patients with severe NCS involvement. Positive results on the clinical battery (Phalen, Tinel, and FDT) could help to confirm the CTS diagnosis, showing a very high specificity and LR+. On the contrary, the low sensitivity is not able to rule out CTS in individuals with negative results.

## Background

Carpal tunnel syndrome (CTS), caused by median nerve entrapment at the wrist, is one of the most common occupational diseases affecting the hand. Its prevalence in the United States working population is 7.8% [1], with higher and increasing incidence in the industrial sector than in the general population [2, 3]. Classic CTS symptoms and sensorimotor alterations include nocturnal pain associated with tingling, numbness, and - in more severe cases - weakness in the median nerve territory that affects the hand motor control, especially during activities involving the first three fingers [4, 5].

A thorough history and physical examination are the key to diagnosis [4]. Thenar atrophy [6] and sensory loss [7] are common findings, but not always present in the early stages of the disease [4, 8] and unable to rule out CTS [9]. Provocative tests aimed at reproducing the patient’s symptoms - such as the Phalen’s test and Tinel’s sign - are often used in the clinical setting, but there is no evidence to support their use as independent diagnostic tools [9]. Moreover, these tests address only the component of Body Functions & Structures of human functioning within the ICF framework [8, 10]. There is conflicting evidence on the diagnostic accuracy even of other more recent tests such as the upper-limb neurodynamic tests or the Flick Sign [4, 9, 11] or of imaging modalities, e.g. ultrasound [12].

Moderate evidence supports the use of nerve conduction studies (NCS) to aid the diagnosis of CTS [9], although a massive use of electrophysiological testing has been strongly criticized. In 2013, the Royal College of Surgeons of England recommended to use NCS only in secondary care for equivocal clinical examination and history, in persistent or recurrent CTS, or in cases of an unclear diagnosis suggesting peripheral neuropathy [13].

The prevalence proportion of CTS reported in cross-sectional studies varies depending on the diagnostic method used - i.e. by median nerve symptoms alone (15%), or by NCS alone (31%), or by both symptoms and NCS (7%) [1] - highlighting the urgent need for a diagnostic reference standard [9].

To be clinically useful, a diagnostic test should be valid, reproducible, easily performed, and have high sensitivity and specificity. If available tests do not reach the required accuracy, then a combination of tests will be more accurate. However, to maximize the accuracy of the battery, the included tests should be conditionally independent (i.e. tapping different aspects of an illness), and not susceptible to patient input. Subjects with CTS frequently have a reduced performance in work, hobbies, and daily living activities, especially when hand dexterity is a required component such as in writing, manipulating small objects or executing the reach-to-pinch maneuver [14–16]. Dexterity is also an important outcome [17], and its assessment via pegboard tests may be of use in the diagnostic process. The pegboard tests are objective, can be easily blinded to the results of other diagnostic procedures, and are fully independent of provocative tests. The test falls under the Body Function (b760d control of voluntary movement functions) and Activity and Participation (d440d fine hand use) ICF domains with a focus on capacity [18, 19], and meets also the demands that functional performance-based measures are included that are not only pain dependent.

A recent pilot study reported a significant correlation (Spearman rho = 0.48, *p* <  0.001) between the Functional Dexterity Test (FDT) and electrophysiological classification [20]. However, these findings need to be interpreted with caution due to a spectrum bias in the cohort (the severe-to-extreme CTS group was under-represented) and the use of penalty scores, which may have reduced the sensitivity of the test to measure in-hand manipulation [18, 21]. Therefore, the use of the FDT to discriminate between subjects needs to be confirmed in a well-balanced sample of individuals with classical CTS symptoms and using the net scores without the penalty system.

The aims of this study were: i) to examine the relationship between fine manual dexterity and NCS in workers with classic CTS symptoms; ii) to investigate the ability of the FDT to discriminate among different CTS severity subgroups as classified by NCS, and iii) to explore the diagnostic accuracy of a clinical battery composed of the FDT and two commonly used provocative tests, using the NCS as reference standard.

## Methods

### Study design

Primary clinical measurement, cross-sectional study. The study was conducted using the STARD 2015 checklist [22].

### Participants and setting

Subjects referred to the Istituti Clinici Scientifici Maugeri to undergo NCS were consecutively screened and recruited for the study until the required clustered sample size - i.e. at least 30 subjects for each group - was reached.

Individuals were eligible if they were referred by a physician with suspected CTS (either unilateral or bilateral), led an active working life, and were of age ranging from 20 to 69 years. People with comorbidities affecting manual dexterity such as fractures or surgical procedures at the upper limb, cervical whiplash in the past 3 months, finger amputations, polyneuropathies/systemic neurological conditions, CTS recurrence, trapeziometacarpal osteoarthritis, tenosynovitis of wrist and fingers, in state of pregnancy, or under concurrent treatment with neurotoxic medications (chemotherapy), were excluded from the study.

To limit selection and spectrum bias, no asymptomatic control group was included, and the convenience sample had to have adequate cluster distribution for CTS severity. CTS severity was classified into four different levels based on NCS results.

The study was approved by the Institutional Review Board of the Istituti Clinici Scientifici Maugeri, and was carried out in compliance with the Declaration of Helsinki. Participants received instruction about the additional tests they would undergo but were not informed about the study purposes. After this, they gave informed written consent to participate in the study.

### Measures

Subjects were screened for eligibility by the same researcher, who did not take part in the evaluation process. The assessment procedure was carried out in three steps with the same order: i) dexterity test, ii) clinical symptoms reproduction tests, and iii) NCS testing. Unilateral versus bilateral cases were recorded from the referring physician’s prescription.

#### Dexterity test

A physical therapist administered the FDT in accordance with the original instructions [23]. The FDT was selected because it is quick to administer, accurate, psychometrically robust [24, 25], and norms for adult workers are available [21]. It provides useful information on the patient’s ability to use the involved hand during daily and work-related activities requiring a 3-point pinch grip. The task consists in turning upside down 16 pegs arranged on a peg board in 4 rows of 4, as quickly as possible using the first three fingers of one hand. It is not allowed to rotate the forearm or to lean the elbow against the table. Subjects were instructed on the procedure and invited to do a trial in order to familiarize themselves with the test. As done in a recent study on children [18], we recorded the net time without considering the penalty system.

#### Clinical symptoms reproduction tests

Two expert physical therapists (> 10 years of practice), blinded to the FDT results, independently administered the Phalen’s test and Tinel’s sign [26, 27]. In Phalen’s test, participants were asked to sit and rest their elbows on a table while holding both forearms in vertical alignment, with the volar surfaces aligned medially. Participants were then instructed to let their wrists relax into full palmar flexion, and a positive response was defined as the reproduction of symptoms in the median distribution of the palmar hand within 60 s.

Tinel’s sign was performed by tapping on the distal wrist crease over the median nerve with a tendon hammer. A positive response was defined as a sensation of tingling in the distribution of the median nerve in the hand. The evocative tests were judged as positive when both tests gave a positive result [27]. Any discrepancies that emerged between the two physical therapists were resolved with the intervention of a third clinician.

#### NCS testing

Subjects underwent bilateral NCS testing (*Medelec Sapphire Premiere 4, Vickers Medical, Old Woking, UK)*, which is used as a reference standard for the diagnosis of CTS for research purposes [28]. The NCS was performed by the same clinician, blinded to all other clinical test results. Distal motor and sensory latency, and motor and sensory conduction velocity between wrist and fingers were recorded. Hands were divided into classes of severity based on the neurophysiological classification of Padua et al. [29], which provides clear neurophysiological cut-offs (normal/abnormal conduction findings and presence/absence of evoked responses) making the assignment easy and non-arbitrary. Group A (GrA) consisted of patients with extreme or severe alterations (absence of thenar motor responses or absence of sensory response and abnormal distal motor latency); Group B (GrB) indicated moderate alterations (abnormal digit-wrist conduction and abnormal distal motor latency); Group C (GrC), slight or minimal alterations (abnormal digit wrist conduction and normal distal motor latency); and Group D (GrD), normal findings on all tests (segmental and comparative included).

### Statistical analysis

The software package SPSS 20 (SPSS Inc., Chicago, IL, USA) was used for the analysis, and statistical significance was set at 95%.

For concurrent agreement with NCS and discriminative purposes, the FDT scores were standardized according to sex, dominant/non-dominant side, and age group by calculating the ratio between the net scores and normative values from a healthy Italian population [21]. Standardized FDT scores (sFDT) were calculated with the following formula: FDT net score/FDT net score representing the 50th percentile of the matched healthy population – 1 [30]. A ratio equal to 0 indicated that the test execution time was equal to that of the matched healthy population; positive values indicated a worse performance, and negative values a better performance compared to the healthy population.

The non-parametric Spearman test (rho) was used to investigate the correlation between the sFDT scores and the NCS-based classification of CTS severity. The hypothesis was that we would obtain a significant correlation between the sFDT scores and the NCS subgroups. As a rule of thumb, where samples are to be broken into subgroups or categories a minimum sample size of 30 cases per category is necessary [31].

A univariate ANOVA with Tukey’s post-hoc analysis was used to investigate the ability of the sFDT to discriminate among subjects allocated to different subgroups. We expected the test to be able to differentiate two or three groups of subjects. ROC curve analysis was also performed to explore the accuracy of the score and to set the cutoff points that best discriminated between groups. Accuracy was measured by the area under the curve: an area of 1 represented a perfect test, while an area of 0.5 represented a worthless test. Cutoff scores were set at two levels: one representing the best trade-off between sensitivity and specificity (Younden index), the other favoring specificity, setting the sensitivity not lower than 50%.

To calculate diagnostic accuracy of the clinical battery (composed of provocative tests plus FDT net score), data were formatted into standard two-by-two tables. The response to the battery was considered positive if all three tests were positive. Subjects with at least minimal NCS alterations were classified as positive, while those with normal findings (GrD) were classified as healthy. If there were no false positive results recorded, the table was adjusted by adding 0.5 to each cell, given that the absence of false positive findings would produce a specificity value of 1 [10]. As a general rule, clinicians would assume this condition likely to be present when a test is positive and the test has a high specificity; conversely, they would assume it to be absent when a test is negative and the test has a high sensitivity [32]. When using a continuous scale, the scores need to be dichotomized and information about the usefulness of the test may be lost. The choice of the threshold used for dichotomization influences the sensitivity and specificity: when sensitivity rises, specificity falls. Hence, different thresholds were explored to find the most useful one. A positive result was considered when the FDT net score was higher than the normative value at a specified percentile, matched to sex, dominant/non-dominant side, and age group. Data from three different thresholds were available for norms (50th, 84th, and 97.5th percentile) [21], allowing to dichotomize the FDT scores at four levels: i) > 97.5th percentile; ii) >84th percentile; iii) >50th percentile; and iv) ≤ the median scores. Sensitivity, specificity, and likelihood ratios (LRs) were calculated, with their respective confidence intervals (CI) with a probability of 95%. The LRs combine the benefits of both sensitivity and specificity into one index, and have the advantage that they can be applied to score intervals for tests with continuous measures. LRs were interpreted as suggested by Jaeschke et al. [33] (Table 1): LR+ greater than 1 increases the likelihood that the target disorder is present, whereas LR- less than 1 decreases this likelihood.

**Table 1:** A guide to interpretation of Likelihood Ratio (LR) values (Adapted from Jaeschke, 1994). Legend: LR + = positive likelihood ratio; LR- = negative likelihood ratio

LR+	LR-	Interpretation
> 10	< 0.1	Generates large and often conclusive shifts in probability.
5–10	0.1–0.2	Generates moderate shifts in probability.
2–5	0.2–0.5	Generates small, but sometimes important, shifts in probability.
1–2	0.5–1	Alters the probability to a small, and rarely significant, degree.

## Results

### Sample characteristics

Among the 152 patients referred for neurophysiological investigations for suspected CTS, 141 (104 females, 37 males) fulfilled the eligibility criteria and were enrolled in the study. Of these, 102 participants reported unilateral symptoms and 39 bilateral symptoms, providing a sample of 180 hands. Recruitment stopped when the sample size target was reached. According to the NCS results, 32 hands (18%) had normal findings and were classified as negative (GrD), while the remaining 148 hands (82%) showed abnormal nerve conduction, and were allocated to one of three different severity groups (GrA = 30; GrB = 71; GrC = 47). Demographic data and sFDT scores of the sample are presented in Table 2. There were no missing data, and no adverse events observed in this study.

**Table 2:** Demographic characteristics of the sample. Legend: GrA, severe/extreme carpal tunnel syndrome; GrB, moderate carpal tunnel syndrome; GrC, mild/minimal carpal tunnel syndrome; GrD, negative carpal tunnel syndrome; sFDT, standardized FDT net scores

	GrA	GrB	GrC	GrD	All
**Hands, N**	30	71	47	32	180
**Women (Hands), N (%)**	20 (66.7%)	51 (71.8%)	40 (85.1%)	20 (62.5%)	131 (72.8%)
**Men (Hands), N (%)**	10 (33.3%)	20 (28.2%)	7 (14.9%)	12 (37.5%)	49 (27.2%)
**Age, mean ± SD**	56.1 ± 10.5	53.9 ± 9.5	47.5 ± 10.2	47.7 ± 7.6	51.8 ± 10.8
**Dominant hands, N (%)**	21 (70%)	43 (60.6%)	22 (46.8%)	21 (65.6%)	107 (59.5%)
**sFDT, mean ± SD**	0.36 ± 0.26	0.17 ± 0.30	0.17 ± 0.26	0.09 ± 0.17	0.19 ± 0.27

### Relationship and discriminative ability

**Fig. 1:**
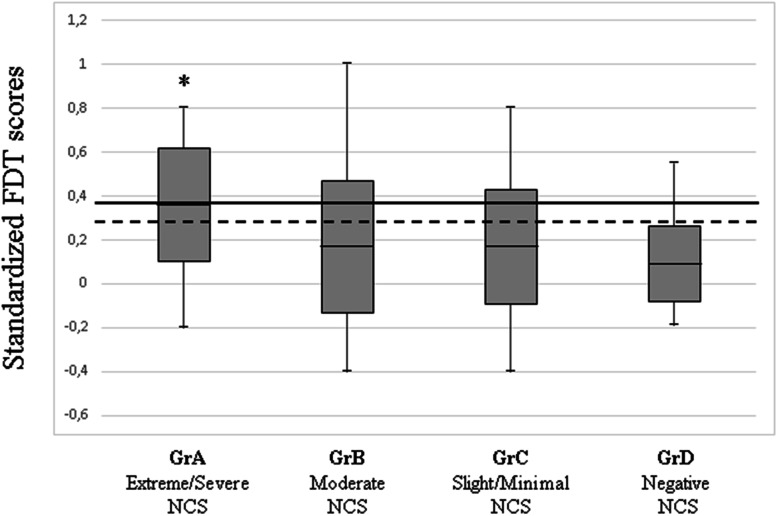
Box-plot distribution of the standardized FDT scores in the diagnostic categories based on NCS. Legend: * = *p* < 0.01; solid line: represents the cutoff (0.36) that favors specificity, setting the sensitivity at a level not lower than 50% in the ROC curve; dotted line: cutoff (0.29) representing the best trade-off between sensitivity and specificity in the ROC curve

Overall, the sFDT scores correlated significantly (Spearman’s rho = 0.25, *p* < 0.001) with the NCS findings. The difference between subgroups was significant (F = 5.942, *p* = 0.001). The Tukey post hoc test revealed that the standardized time to complete the FDT was lower in GrB (*p* = 0.01), GrC (*p* = 0.005) and GrD (*p* = 0.001) than in GrA, but no other significant differences were observed (Fig. 1).

**Fig. 2:**
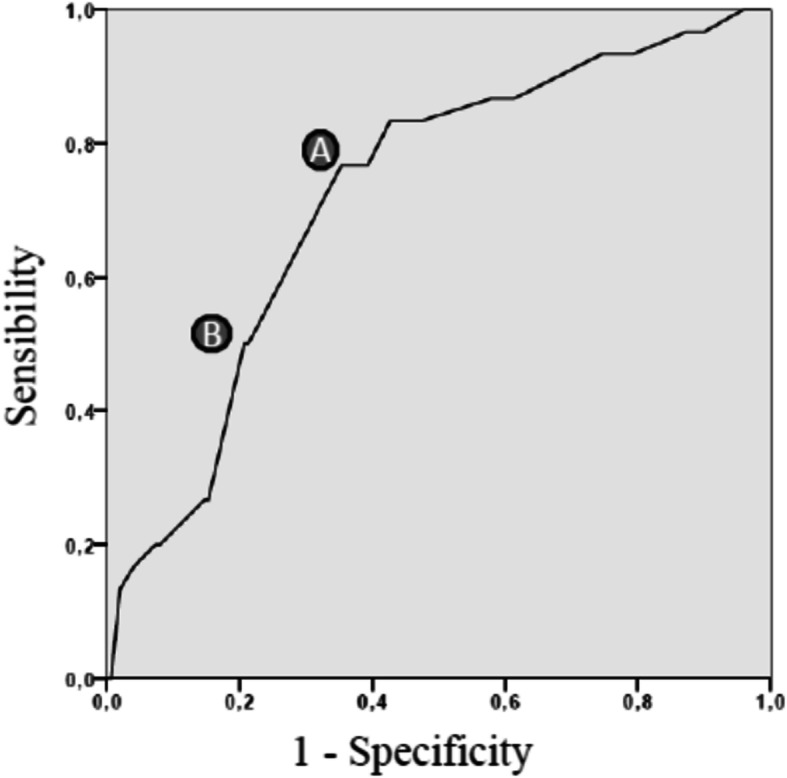
ROC curve. Legend: A = cutoff representing the best trade-off between sensitivity and specificity; B = cutoff that favors specificity, setting the sensitivity at a level not lower than 50%

A ROC curve was plotted to determine the accuracy of the sFDT scores to discriminate subjects with extreme/severe NCS alterations (GrA). The accuracy was fair, with an area under curve of 0.72 (95%CI: 0.63–0.82). The two different cutoff points were set at 0.29 (sensitivity: 77%, specificity 65%), and 0.36 (sensitivity: 50%, specificity 80%) (Fig. 2).

### Diagnostic accuracy

**Fig. 3:**
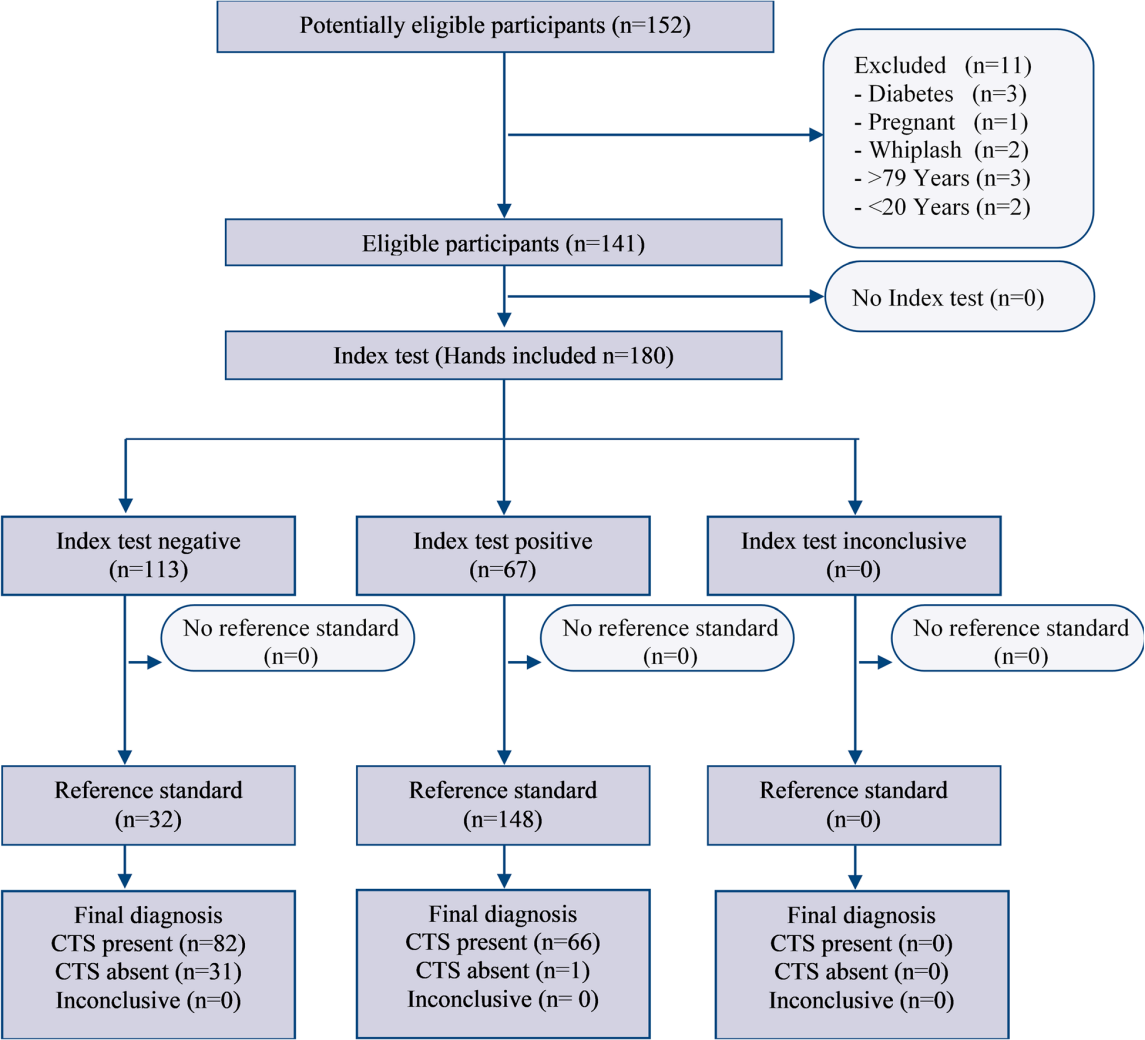
Flowchart of the diagnostic accuracy study. The Reference standard was the Nerve Conduction Studies (NCS), while the Index test was the clinical battery composed of the Functional Dexterity Test (FDT), Phalen’s test and Tinel’s sign. In this chart, the FDT net scores threshold was set at >84th percentile of the normative values

In our study cohort, the thresholds used to consider a positive FDT yielded a highly variable level of sensitivity (ranging from 18 to 77%) and high specificity (91 to 98%) for the whole clinical battery (Table 3). Figure 3 shows a flow chart of the diagnostic accuracy study, with the FDT net scores threshold set at >84th percentile of the normative values.

**Table 3:** Accuracy values of the battery including the sFDT (set at four different thresholds), Phalen’s, and Tinel’s tests for each of the cut-off values. Legend: SENS = sensitivity; SPEC = specificity; LR + = positive likelihood ratio; LR- = negative likelihood ratio; CI = confidence interval

	SENS (± CI_**95%**_)	SPEC (± CI_**95%**_)	LR+ (± CI_**95%**_)	LR- (± CI_**95%**_)
**FDT score > 97.5th percentile**	0.18 (0.12–0.25)	0.98 (0.87–1.00)	11.74 (0.73–187.76)	0.84 (0.77–0.91)
**FDT score > 84th percentile**	0.45 (0.37–0.53)	0.97 (0.83–0.99)	14.49 (2,09 - 100,53)	0,56 (0,48 - 0,66)
**FDT score > 50th percentile**	0.63 (0.55–0.70)	0.91 (0.76–0.97)	6.70 (2.27–19.82)	0.41 (0.32–0.52)
**FDT score ≤ 50th percentile**	0.77 (0.70–0.83)	0.91 (0.76–0.97)	8.22 (2.79–24.21)	0.25 (0.19–0.35)

## Discussion

The first aim of the study was to investigate the correlation between the fine manual dexterity measured through the FDT and NCS findings in individuals presenting classical CTS symptoms. Among the several standardized tests developed to assess manual dexterity, the FDT was chosen because it uses a three-digit pinch to investigate dynamic dexterity, including both prehension and fine manipulative abilities [23]; it also received the best ratings for clinimetric quality [25], and reference norms were available in the healthy population [21]. In our study, the penalty system (5 and 10 s in the case of peg-dropping and non-standardized movements, respectively) was not adopted because it was found to be poorly correlated to the net time*,* and could negatively influence the sensitivity of the test [21].

Our study hypothesis, that sensorimotor impairments affecting hand dexterity would be moderately associated with disease severity, was confirmed (Spearman rho = 0.25, *p* < 0.001). Our results indicated that the dexterity test can discriminate subjects with severe/extreme NCS findings from those with less severe neuropathy. This ability may be of use also during outcome assessment, e.g. to monitor the effect of treatment and the course of recovery in severely affected patients. Furthermore, the FDT may facilitate clinical decision making because it focuses on the hand performance. The two different sFDT cutoff points could be interpreted as the lower and upper boundaries for a small range of reasonable confidence. The lower one, representing the best trade-off between sensitivity and specificity, was set at 0.29 (sensitivity: 77%, specificity: 65%) while the upper one, representing the cutoff point favoring specificity, was set at 0.36 (sensitivity: 50%, specificity: 80%). Clinically, the use of the higher threshold would produce fewer false positives. This means that less people with no, mild, or moderate NCS involvement would be incorrectly identified as having severe CTS, but a higher proportion of people with a negative test would probably be misclassified as healthy or less severely involved. The opposite occurs when using the lower threshold. The principal factor influencing the clinician’s choice of one or other cutoff point is related to the consequence of misclassifying patients [32]. For example, the use of a higher threshold could avoid a large number of unnecessary surgical interventions, while the lower threshold would be more indicated when deciding who should undergo NCS testing or when one wants to reduce the risk of a too-early discharge in the clinical context. In any case, the FDT at a certain point should not be used alone to determine the treatment choice. To make treatment decisions it would be more useful to consider progress and stage of the disease, possibly combining FDT and NCS.

The third aim of this study was to investigate the diagnostic accuracy of the clinical test battery composed of Phalen’s test, Tinel’s sign and the FDT. The reason for including the pegboard test in the battery was to broaden the spectrum of ICF components measured, in line with the Brief ICF Core Set for Hand Conditions suggestions [34, 35]. The key point of the analysis was to set the best threshold to discriminate positive vs. negative FDT scores. The performances recorded in this study were compared to those of matched asymptomatic individuals, and diagnostic accuracy was explored using the available normative thresholds. Setting the threshold at the 97.5th percentile of normative values resulted in no false positives for subjects without NCS abnormalities. The associated LR+ indicated that a positive battery would generate a large post-test probability of CTS, although the large confidence intervals suggested that the probability was not conclusive. On the other hand, a negative test changed the probability of ruling out a subject to a small, and rarely important, degree, with a very large proportion of patients with positive NCS that would not have been further investigated (or treated) if the battery was the only diagnostic method used.

A slightly less restrictive threshold (>84th percentile) would produce a similar specificity (97%) with only one hand misclassified as false positive. This threshold was associated with the highest LR+ value (14.5), and the lower bound of CI > 2 indicated that the positivity of tests would generate at least a small, but sometimes important, shift in the probability to correctly identify subjects with electrodiagnostic impairment. However, false negatives were still observed in a high proportion of subjects, not allowing to rule out subjects with greater precision than chance.

The two thresholds set at >50th and ≤ 50th percentile added no to very low clinical utility over the use of provocative tests alone. The addition of the FDT did not help the clinician to exclude a significant proportion of misclassified hands. Both thresholds produced a specificity of 91%, with the higher limit of CI that only equals the mean threshold value at >84th percentile. Consequently, the shift in odds favoring the condition when the battery was positive was greatly reduced (LR+ of 6.70 and 8.22, respectively). Since there were no individuals that scored under the 50th percentile, the diagnostic accuracy of this threshold could be attributed exclusively to the provocative tests. Within the literature there are many studies investigating the diagnostic values of provocative tests, showing a high variability and often contrasting results. Variations of the same order of magnitude have been reported for both Phalen’s test and Tinel’s sign for both specificity and sensitivity, with values ranging from 30 to 100% and 10–91%, respectively [8, 26, 36–40]. These discrepancies have been attributed to clinical, methodological and statistical factors including a referral bias, the small number of patients examined, different diagnostic criteria of CTS, and different characteristics of control subjects [39]. Some studies have investigated the diagnostic accuracy of combined tests, but the advantage with respect to the tests taken individually was negligible [36, 41–43]. In the present study, the combination of evocative tests produced a high specificity (91%) but only moderate sensitivity (77%), in line with previous data.

The FDT net scores with a threshold set at >84th percentile of the normative values matched by dominant/non-dominant side, age and sex proved to be the best solution to raise the level of specificity (97%) and LR+ (14.5) of the diagnostic battery. Such an approach would be consistent with the intention to adopt diagnostic methods that favor specificity, in order to avoid including subjects without the condition. Otherwise, the proposed battery has very little ability to rule out subjects with negative clinical tests. This was probably attributable to the fact that some subjects may not have performed the FDT to their full potential, obtaining a score higher than their peers. Higher sensitivity (77%; IC 70–83%) was then observed for the provocative tests without the addition of the FDT.

One of the strengths of this study is that only subjects with symptoms of CTS and referred for NCS were included, with quite a high number of hands evaluated. The findings are even more valuable since NCS was available for all those included, and electrodiagnostic findings spanned a large spectrum. Furthermore, the slightly restrictive selection criteria allowed us to enroll a population that can be considered as representative of the real world of patients undergoing NCS. However, this may have raised the pretest probability of detecting CTS rather than studying the general population visiting a physician. In addition, all subjects were recruited from the same geographic region. Therefore, the findings must be interpreted with caution taking into account these selection biases.

The main limitation of the study was that no information regarding symptom duration or other potential risk factors was gathered, preventing further analysis. For instance, it was not possible to determine whether or not the dexterity deficit progressed with time as other physical signs do, i.e. sensation, thenar muscle wastage, etc. Similarly, a possible correlation between hand dexterity and comorbidities could not be investigated.

## Conclusion

This study supports the hypothesis that individuals with CTS suffer sensorimotor impairments that affect hand dexterity. The sFDT was able to discriminate subjects with severe NCS involvement. The clinical battery composed of the Phalen’s and Tinel’s provocative tests combined with a FDT net score above the 84th percentile of matched healthy subjects showed a high specificity and positive LR. This means that positive results could be useful for CTS diagnosis, but the low sensitivity was not sufficient to rule out CTS in those subjects with negative test results.

## Data Availability

The datasets generated and/or analysed during the current study are not publicly available, but are available from the corresponding author on reasonable request.

## Abbreviations

CI: Confidence intervals
CTS: Carpal tunnel syndrome
FDT: Functional Dexterity Test
sFDT: Standardized Functional Dexterity Test score
ICF: International Classification of Functioning, Disability and Health
LR: Likelihood ratio
NCS: Nerve conduction studies
ROC: Receiver Operating Characteristic
STARD: Standards for Reporting Diagnostic accuracy studies
